# Phosphatidylinositol 4‐kinase as a target of pathogens—friend or foe?

**DOI:** 10.1002/1873-3468.70216

**Published:** 2025-11-11

**Authors:** Ana C. Mendes, Guilherme M. Azevedo, Amanda P. Barcellos, Diana Bahia

**Affiliations:** ^1^ Departamento de Genética, Ecologia e Evolução, Instituto de Ciências Biológicas Universidade Federal de Minas Gerais Belo Horizonte Brazil

**Keywords:** inhibitors, pathogens, phosphatidylinositol 4‐kinases, phosphoinositides, PI4K, PI4P, signalling

## Abstract

Phosphatidylinositol 4‐kinases (PI4Ks) are pivotal enzymes responsible for the generation of phosphatidylinositol 4‐phosphate (PI4P), a key precursor involved in various cellular signalling pathways that regulate vesicular trafficking, Golgi maintenance and intracellular communication. Conserved across eukaryotic species, PI4Ks exist in distinct isoforms with specific localisations and functions, ranging from plasma membrane dynamics to endosomal trafficking and autophagy regulation. Notably, numerous pathogens, including viruses, bacteria and parasites, have evolved mechanisms to hijack host PI4Ks, thereby facilitating intracellular replication and survival. For example, RNA viruses within the *Flaviviridae* and *Coronaviridae* families recruit PI4Ks to remodel host membranes for the assembly of replication complexes. Similarly, intracellular bacteria such as *Salmonella* and *Legionella* manipulate PI4P‐enriched compartments to evade host defences and promote replication. Due to their central roles in both normal cellular physiology and infection, PI4Ks have emerged as promising therapeutic targets. A variety of inhibitors, including ATP‐competitive compounds, have been developed and evaluated for their antiviral, antibacterial and antiparasitic potential. Nevertheless, challenges related to selectivity and toxicity remain. Recent advances include the identification of inhibitors effective against *Plasmodium* species and the development of compounds targeting PI4KIIIβ in infections with viruses such as hepatitis C and coronaviruses. This review highlights the dual role of PI4Ks as essential cellular regulators and exploitable pathogen cofactors, underscoring their potential as drug targets. Continued investigation into the structure, function and inhibition of PI4Ks may enable the development of selective therapeutic strategies for infectious diseases while minimising off‐target effects on host cells.

## Graphical abstract

This graphical summary illustrates the roles of phosphatidylinositol 4‐kinases (PI4Ks). PI4Ks regulate key cellular processes and can be hijacked by pathogens, such as viruses, bacteria and parasites, to support their intracellular replication. Their dual role as essential host enzymes and pathogen cofactors makes them promising drug targets. Recent advances have highlighted the potential of selective PI4K inhibitors with antiviral, antibacterial and antiparasitic properties, although challenges relating to toxicity and specificity remain.

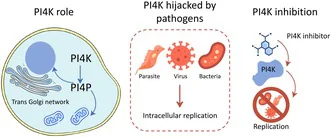

## Abbreviations

**AKT**, protein kinase B

**AP‐3**, adaptor protein complex 3

**Arf 1**, ADP‐ribosylation factor 1

**ARM**, armadillo repeat

**ATG2**, autophagy‐related protein 2

**ATP**, adenosine triphosphate

**CVB3**, Coxsackie B3

**DAG**, diacylglycerol

**EE**, early endosome

**ER**, endoplasmic reticulum

**GAS**, *Streptococcus pyogenes*

**GcAVs**, Group A *Streptococcus*‐containing autophagosome‐like vacuoles

**HCoV**, coronavirus

**HCV**, hepatitis C virus

**HRV1**, human rhinovirus 1

**HRV14**, human rhinovirus 14

HRV2, human rhinovirus 1

**IP**_**3**_, 1,4,5‐trisphosphate

**ISG**, immature secretory granule

**LKU**, lipid kinase unique

**LYS**, lysosome

**MCSs**, membrane contact sites

**MVB**, Multivesicular body

**OMM**, outer mitochondrial membrane.

**ORPs**, oxysterol‐binding protein (OSBP)‐related proteins

**OSBP**, oxysterol‐binding protein

**PH**, pleckstrin homology

**PI**, phosphatidylinositol

**PI3,4,5,P**_3_, phosphatidylinositol 3,4,5‐trisphosphate

**PI3,4P**_**2**_, phosphatidylinositol 3,4‐bisphosphate

**PI3,5P**_**2**_, phosphatidylinositol 3,5‐bisphosphate

**PI3K**, phosphatidylinositol 3‐kinase

**PI3P**, phosphatidylinositol 3‐phosphate

**PI4,5P**_**2**_, phosphatidylinositol 4,5‐bisphosphate

**PI4Ks**, phosphatidylinositol 4‐kinases

**PI4P**, phosphatidylinositol 4‐phosphate

**PI5P**, phosphatidylinositol 5‐phosphate (PI5P)

**PIP**_**2**_, PI 4,5‐bisphosphate

**PIPs**, phosphoinositides

**PKC**, protein kinase C

**PKD**, protein kinase D

**PLC**, phospholipase C

**PS**, phosphatidylserine

**RNA**, ribonucleic acid

**SARS‐CoV‐2**, severe acute respiratory syndrome coronavirus 2

**SCVs**, Salmonella‐containing vacuoles

**SopF**, Specific Opportunist Pathogen Free

**TGN**, Trans‐Golgi network

Eukaryotic cells are compartmentalised into organelles with specialised functions, coordinated by signalling proteins and phospholipids such as phosphatidylinositol (PI). Although PIs constitute only a small fraction of cellular phospholipids, they play critical roles in regulating the recruitment and activity of numerous signalling proteins at membrane surfaces.

PIs consist of a glycerol backbone linked to two hydrophobic acyl chains and a myo‐inositol head group. The inositol ring contains five hydroxyl groups that can be phosphorylated at the D‐3, D‐4 and D‐5 positions. The phosphorylation patterns at these positions, and their combinations, give rise to seven known species of phosphatidylinositol phosphates or phosphoinositides (PIPs): phosphatidylinositol 3‐phosphate (PI3P), phosphatidylinositol 4‐phosphate (PI4P), phosphatidylinositol 5‐phosphate (PI5P), phosphatidylinositol 3,5‐bisphosphate (PI3,5P_2_), phosphatidylinositol 4,5‐bisphosphate (PI4,5P_2_), phosphatidylinositol 3,4‐bisphosphate (PI3,4P_2_) and phosphatidylinositol 3,4,5‐trisphosphate (PI3,4,5P_3_). These PIPs are essential regulators of a wide range of cellular processes. Acting as second messengers, they transmit signals to downstream effectors by recruiting specific proteins through interactions with lipid‐binding domains [1, 2].

Each PIP species is distributed in variable amounts among cellular compartments, where they regulate processes such as membrane trafficking, cytoskeletal dynamics, the activity of integral membrane proteins, plasma membrane signalling, endosome dynamics and Golgi function [3, 4]. PIPs are derived from PI, which originates in the endoplasmic reticulum and can be rapidly interconverted by the coordinated action of lipid kinases and phosphatases, allowing for the tightly regulated spatiotemporal control of signalling. This capacity renders them ideal regulators of dynamic cellular events, particularly in response to physiological stimuli requiring rapid signalling [5].

PI4P is a member of the phosphoinositide family that serves as a key precursor in the PIP signalling pathway. PI4P is primarily synthesised by phosphatidylinositol 4‐kinases (PI4Ks) and constitutes the initial substrate for the generation of PI 4,5‐bisphosphate (PIP_2_) and PI 3,4,5‐trisphosphate (PIP_3_), two major phosphoinositides involved in cell signalling cascades [5]. PIP_2_ is cleaved by phospholipase C (PLC) to generate the second messengers inositol 1,4,5‐trisphosphate (IP_3_) and diacylglycerol (DAG), both of which are crucial mediators of calcium‐dependent signalling. These messengers activate downstream pathways via protein kinase C (PKC) to contribute to processes such as growth factor signalling, cytokine induction, neurotransmitter release and muscle contraction. Phosphorylation of PIP_2_ by class I PI3K generates PIP_3_, which activates Akt‐PKB and other signalling proteins involved in cell survival and proliferation [6].

This review emphasises the dual role of PI4Ks as essential cellular regulators and exploitable pathogen cofactors, highlighting their potential as drug targets.

### The cellular roles of phosphoinositides and phosphatidylinositol 4‐kinases

PI4P also functions as a major regulatory lipid at the Golgi apparatus, trans‐Golgi network (TGN) and certain endosomal and vesicular compartments. Its highest steady‐state concentrations are found in the plasma membrane and the membranes of the Golgi. The Golgi apparatus plays a central role in glycoprotein maturation and protein sorting for delivery to the endosomal system or for exocytosis. It also contributes to lipid biosynthesis, serving as a site for the generation of various sphingolipid species [2, 7].

All eukaryotic organisms possess two conserved families of PI4Ks: type II and type III. These families differ in domain organisation and functional characteristics. Type III PI4Ks are referred to as ‘typical’ kinases due to their structural similarity to PI3Ks, while type II PI4Ks are considered ‘atypical’ as they share no homology with other lipid kinases. The originally identified type I PI4K, isolated by chromatographic methods from bovine brain tissue, was later reclassified as PI3K [8].

**Fig. 1:**
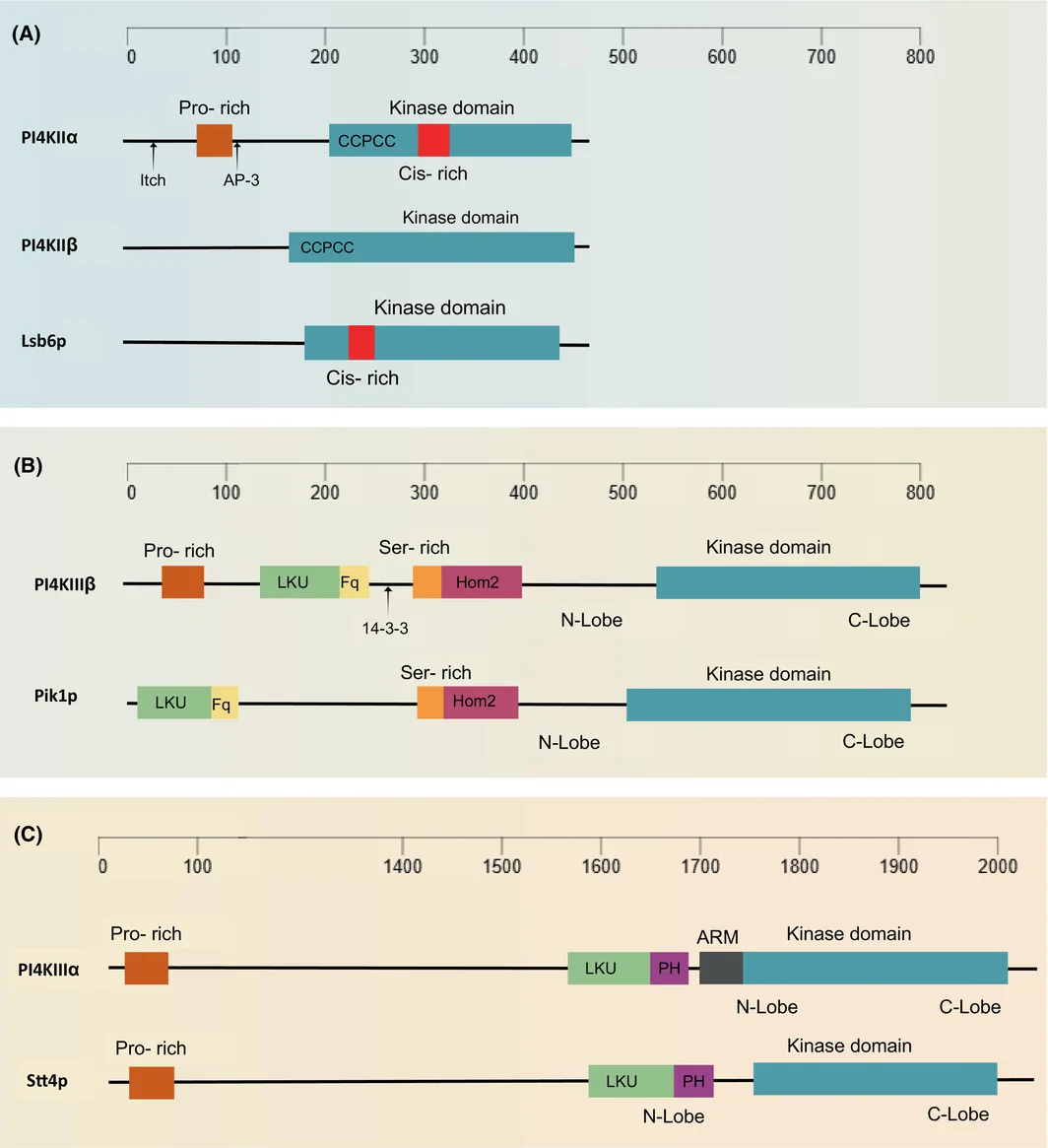
(A) PI4KIIα and PI4KIIβ exhibit a high degree of structural similarity and share greater homology with Ser/Thr protein kinases than with other lipid kinases. They are relatively small proteins, and their kinase domains display limited sequence similarity to those of type III PI4Ks. Both type II isoforms contain a cysteine‐rich catalytic motif (CCPCC), which undergoes palmitoylation, a post‐translational modification involving the covalent attachment of palmitic acid. This palmitoylation event is a key regulatory mechanism for type II PI4Ks. The kinase domain of PI4KIIα is located at the C terminus and is preceded by a short flexible α‐helix. It includes two stretches that are conserved from yeast to humans. Within the catalytic domain, the CCPCC motif serves as the site of palmitoylation. In addition, PI4KIIα contains binding sites for the E3 ubiquitin ligase Itch and for the clathrin adaptor protein complex (AP‐3) within its proline‐rich N‐terminal region. This amphipathic N terminus also mediates interaction with AP‐3 via an AP‐3 binding motif [9, 10]. PI4KIIβ similarly possesses an unstructured N terminus, lacking the flexible α‐helix found in PI4KIIα, but includes a highly acidic region that is proline‐rich. The hallmark feature of the type II kinase domain – the palmitoylated CCPCC motif – is retained and functional *in vivo*. The acidic N‐terminal domain of PI4KIIβ facilitates interaction with adaptor protein 1. Lsb6p, the type II PI4K in *S. cerevisiae*, contains a C‐terminal kinase domain embedded within a cysteine‐rich region [3, 11]. (B) PI4KIIIβ features a lipid kinase unique (LKU) domain at the N terminus that is predicted to adopt a helical structure. Adjacent to the LKU domain lies the frequenin‐binding site. This kinase includes three intrinsically disordered regions: the N terminus, which binds ACBD3; a loop between the LKU and kinase domains, which serves as a 14‐3‐3 protein‐binding site; and a disordered loop within the N lobe of the kinase domain with unknown function. PI4KIIIβ also contains a serine‐rich region and notably lacks a pleckstrin homology (PH) domain [12, 13]. Pik1p, the yeast orthologue of PI4KIIIβ, harbours a C‐terminal kinase domain and an N‐terminal LKU domain that includes a frequenin‐binding site. Conservation of this domain architecture is evident between Pik1p and vertebrate PI4KIIIβ. Pik1p also possesses a region (Hom2) with sequence similarity to the Rab‐binding domain found in mammalian and *Arabidopsis* PI4KIIIβ isoforms. (C) PI4KIIIα comprises an N‐terminal LKU domain followed by a PH domain, located between the LKU and catalytic domains. It also contains a proline‐rich region. The kinase domain is situated at the C terminus and is followed by an armadillo repeat (ARM) domain and three predicted helical subdomains [14].

Four distinct PI4K isoforms have been identified in mammalian cells: two type II enzymes named PI4KIIα (55 kDa) and PI4KIIβ (55 kDa), and two type III enzymes named PI4KIIIα (230 kDa) and PI4KIIIβ (92 kDa). In *Saccharomyces cerevisiae*, three conserved PI4Ks have been characterised: Stt4p, the orthologue of PI4KIIIα; Pik1p, the orthologue of PI4KIIIβ; and a single type II PI4K, Lsb6p. These enzymes have also been identified in *Drosophila melanogaster* [3, 6] (Fig. 1).

In addition to their distinct biochemical properties, PI4Ks exhibit diverse cellular functions. In *S. cerevisiae*, Stt4p is essential for yeast viability and regulates actin integrity and organisation at the plasma membrane. Pik1p, also essential, localises primarily to the Golgi apparatus, where it controls secretory trafficking. It is also capable of translocating to the nucleus, indicating additional roles in secretion. In contrast, Lsb6p, which is implicated in actin regulation, polymerisation and endosomal mobility, is nonessential for yeast survival. Type II PI4Ks are distributed across nearly all cellular membranes. In humans, PI4KIIα is the most catalytically active isoform, being responsible for synthesising ~50% of total PI4P. It localises primarily to the Golgi and endosomal membranes and functions in vesicle formation and endosome trafficking [3, 6]. PI4KIIα has been identified as the primary enzyme responsible for PI4P production at lysosomes, and studies have demonstrated its pivotal function in lysosomal repair. PI4KIIα is responsible for synthesising PI4P, the initial signalling lipid that marks damaged lysosomes. Lysosomal PI4P has been shown to recruit several ORPs (oxysterol‐binding protein (OSBP)‐related proteins), including ORP9, ORP10 and ORP11, to establish new and extensive endoplasmic reticulum–lysosome membrane contact sites (MCSs). This process has been demonstrated to mediate the transport of phosphatidylserine (PS) and cholesterol from the ER to lysosomes. The process of PS accumulation within lysosomes has been shown to activate the lipid transporter ATG2, which, in turn, delivers substantial quantities of lipids to the lysosomal membrane. This process has been demonstrated to facilitate direct and efficient lysosomal repair [15, 16, 17, 18]. In addition, PI4KIIα plays a regulatory role in Wnt signalling, a pathway critical for tissue development.

**Fig. 2:**
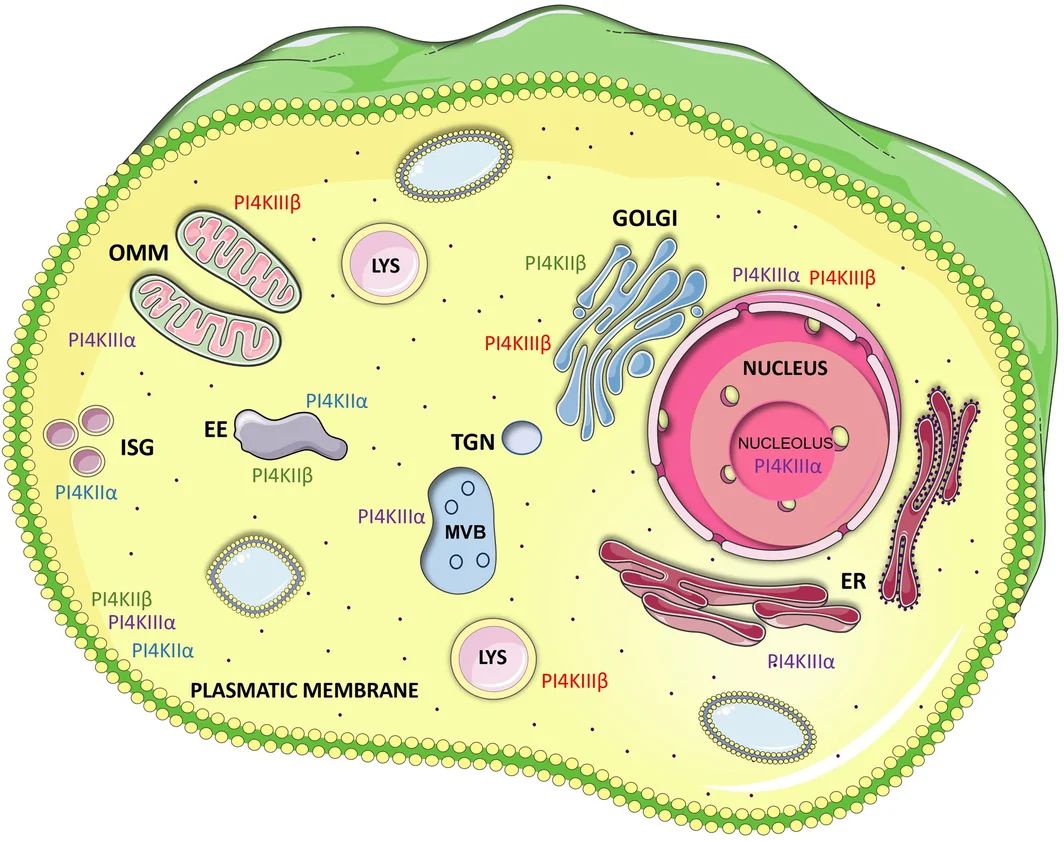
Reported cellular localisations of PI4Ks. This figure summarises the localisation of various PI4K isoforms based on data derived from mammalian cells, *S. cerevisiae* and other animal model systems. Each PI4K is listed adjacent to the organelle with which it is primarily associated. EE, early endosome; ISG, immature secretory granule; LYS, lysosome; MVB, multivesicular body; OMM, outer mitochondrial membrane.

In mammals and *Drosophila*, PI4KIIIα is predominantly associated with the endoplasmic reticulum but is dynamically recruited to the plasma membrane. It has also been detected in the Golgi, nucleus, multivesicular bodies and outer mitochondrial membrane. PI4KIIIα is the primary enzyme responsible for PI4P production at the plasma membrane. Mammalian PI4KIIIβ localises to the Golgi apparatus, nucleus, endoplasmic reticulum, outer mitochondrial membrane and lysosomes (Fig. 2) [3, 6]. It is actively recruited to the Golgi, where it plays a critical role in Golgi transport and serves as a key regulator of vesicular trafficking in the late secretory pathway. The production of PI4P by PI4KIIIβ influences cellular processes such as adhesion, migration and morphology. As observed in yeast, PI4K activity in mammals is essential for maintaining the structural and functional organisation of the Golgi complex [8].

Wang *et al*. [19] demonstrated that Pik1p and Stt4p are required for autophagy in *S. cerevisiae*. Their findings showed that Pik1p regulates Atg9p trafficking from the TGN to the preautophagosomal structure in both selective and nonselective autophagy. Kurokawa *et al*. [20] suggested that Pik1p plays an essential role in autophagosome formation, while Stt4p may primarily generate PI4P at the cytoplasmic leaflet of the autophagosomal outer membrane, thereby promoting autophagosome–vacuole fusion in yeast cells. In a subsequent study, Kurokawa *et al*. [21] reported a significant reduction in PI4P levels within microautophagic vesicles in pik1^ts^ and stt4^ts^ mutant yeast strains, indicating that both Pik1p and Stt4p contribute to microautophagy at the vacuolar membrane.

Hausser *et al*. [22] found that the active protein kinase D (PKD) isoforms PKD1 and PKD2 phosphorylate phosphatidylinositol 4‐kinase IIIβ (PI4KIIIβ) at serine 294, leading to enhanced lipid kinase activity. The authors proposed that PKD regulates secretory transport by phosphorylating proteins involved in vesicular trafficking. PKD comprises a family of serine/threonine kinases (PKD1, PKD2 and PKD3) that participate in a range of biological processes, including cell motility, differentiation, vesicle secretion, gene transcription, innate immunity and cytokine production. Depending on the cell type and external stimuli, PKDs localise to the cytosol, nucleus, Golgi complex and plasma membrane. Recent studies have highlighted a crucial role for PKD in Golgi function, particularly in mediating vesicle fission from the TGN. This kinase is central to the transport of cargo from the TGN to the plasma membrane. Recruitment of PKD to the TGN is initiated by diacylglycerol (DAG) and ADP‐ribosylation factor 1 (Arf1), a small GTPase that acts as both a sensor of the lipid environment and a signal transducer [23, 24].

Given their critical roles in maintaining cellular homeostasis and survival, PI4Ks have been implicated in the pathogenesis of various diseases, including cancer, Alzheimer's disease and bacterial and viral infections. Moreover, PI4Ks in parasitic organisms are promising therapeutic targets for the treatment of infectious diseases such as malaria [25].

### Pathogens subvert cellular PI4ks

PI4Ks play critical roles in various pathological processes. Several RNA viruses sequester PI4KIIIs during the early stages of replication. Members of the *Flaviviridae*, *Picornaviridae* and *Coronaviridae* families exploit host PI4Ks to remodel intracellular membranes, thereby establishing replication complexes. Coronaviruses are enveloped, positive‐sense, single‐stranded RNA viruses that rely on endoplasmic reticulum and Golgi membranes for genome replication [26]. Studies of the coronavirus envelope (E) protein have demonstrated its association with the TGN [27]. Several host proteins are recruited into the coronavirus replication complex, including PI4KIIIβ. The serine/threonine kinase PKD regulates numerous intracellular processes through the phosphorylation of diverse substrates, including PI4KIIIβ [22]. Han *et al*. [28] demonstrated that PKD plays a critical role in coronavirus replication, with the inhibition of PKD impairing replication via a PI4KIIIβ‐dependent signalling pathway. Their findings also showed that the inhibition of PI4KIIβ reduces the replication of common coronaviruses. Collectively, these results indicate that PI4KIIIβ activity is essential for human coronavirus replication and suggest that PI4KIIIβ facilitates viral replication by regulating Golgi membrane fission.

PI4Ks also play significant roles in bacterial infections. Many pathogenic bacteria have evolved an intracellular lifestyle to support their replication while evading detection and destruction by host immune defences. This strategy not only ensures their survival and proliferation but also enhances their virulence [29]. To achieve this, bacterial pathogens manipulate host lipid signalling through the action of secreted bacterial effectors that target membrane‐localisation domains and alter the expression or distribution of specific lipid molecules [30, 31, 32]. Among the most affected lipid species are phosphoinositides, which are enriched at the cytosolic face of cellular membranes and are crucial regulators of signal transduction, cytoskeletal rearrangement and membrane dynamics. These lipids create a highly specialised microenvironment for the recruitment of signalling proteins, initiation of signal cascades and evasion of host immune responses [29, 31]. Lau *et al*. [29] demonstrated that, during *Salmonella* infection, bacterial effectors such as SopF interact with PI4P at the membranes of *Salmonella*‐containing vacuoles (SCVs). This interaction is essential for the stabilisation and maturation of SCVs, providing a permissive niche for bacterial survival and replication. Furthermore, the modulation of PI4P levels within SCVs prevents their fusion with lysosomes, thereby protecting the bacteria from degradation [29; 32].

PI4P has been detected throughout the membrane of SCVs and in associated tubules during *Salmonella enterica* serovar Typhimurium infection in HeLa cells, suggesting that phosphoinositides are key structural and functional components of the SCV system. Inhibition of PI4Ks results in the displacement of SteA, a bacterial effector protein that binds specifically to PI4P, thereby reinforcing its reliance on this phosphoinositide for subcellular localisation [30].

Other intracellular pathogens, including *Chlamydia* and *Legionella*, also exploit PI4P at the membranes of their intracellular vacuoles to recruit host proteins essential for vacuolar membrane remodelling and bacterial replication. In the case of *Legionella pneumophila*, replication is directly dependent on PI4KIII activity, and pharmacological inhibition of this kinase markedly reduces bacterial proliferation [31].

Suppression of PI4KIIα reduces *Listeria monocytogenes* invasion [33] and its depletion impairs vacuole formation required for *Chlamydia trachomatis* replication [34]. In addition, PI4Ks are involved in the autophagic elimination of *Streptococcus* pyogenes (Group A *Streptococcus*). Nakajima *et al*. [35] reported that RAB30, a small GTPase that regulates the TGN, recruits PI4Ks to control PI4P levels. This promotes the formation of Group A *Streptococcus*‐containing autophagosome‐like vacuoles (GcAVs) and facilitates pathogen clearance.

### PI4Ks are a lethal target for parasites

Beyond bacterial infections, PI4Ks have been identified as potential therapeutic targets in parasitic diseases. In *Plasmodium falciparum*, PI4K inhibitors disrupt parasite development at multiple stages of the life cycle, demonstrating that PI4K is essential for cellular regulation and is a promising target for drug discovery and development [36]. Similarly, Rodgers *et al*. [1] reported that PI4KIIIβ in *Trypanosoma brucei* is vital for parasite survival, with depletion of this kinase leading to marked morphological abnormalities and impaired growth. Their study highlighted the importance of PI4KIIIβ in cellular cytokinesis, Golgi maintenance, secretion and the positioning of the nucleus and kinetoplast. Babesiosis is a tick‐borne illness caused by *Babesia* species, including *Babesia microti*. Ji *et al*. [37] demonstrated that PI4K inhibition effectively suppresses parasite growth. The novel PI4K inhibitor MMV390048, originally developed as an anti‐*Plasmodium* compound, exhibits potent activity against *Babesia gibsoni in vitro* and against *B. rodhaini* and *B. microti in vivo*. In a separate study, Ji *et al*. [38] reported that a 64‐day treatment with MMV390048 alleviated babesiosis in severe combined immunodeficiency mice infected with *B. microti*. These results confirm that PI4K is a promising therapeutic target for parasite clearance. Moreover, inhibition of *B. microti* PI4K suppressed parasite growth and eradicated infection in immunocompromised hosts. Collectively, these findings suggest that PI4K inhibitors hold substantial potential for the development of therapeutics against viral, bacterial and parasitic infections (Table 1).

**Table 1:** PI4K's roles in the life cycle of important pathogens and the impacts of its inhibition.

Pathogen	PI4K function in pathogen's life cycle	The impacts of PI4K inhibition	References
*Plasmodium falciparum*	PI4Ks are essential in pathological processes.	Interruption of the parasite's development at multiple stages of the life cycle.	[36]
*Trypanosoma brucei*	PI4KIIIβ is important in cellular cytokinesis, Golgi maintenance, secretion and the correct position of the nucleus and kinetoplast.	Depletion of the kinase results in severe morphological and growth defects.	
*Coronavírus (HCoV)*	PI4KIIIβ is essential for HCoV replication, and PI4KIIIβ can mediate HCoV replication by regulating the fission of cellular Golgi.	PI4KIIβ inhibition decreases common coronavirus replication.	[22, 26, 27, 28]
*Salmonella typhimurium*	Bacterial effectors, such as SopF, bind to PI4P in the membranes of *Salmonella*‐containing vacuoles (SCVs). This binding is essential for the stabilisation and maturation of the SCVs, creating a favourable environment for the bacteria's survival and replication.	—	[29, 32]
*Chlamydia trachomatis*	PI4P is recruited into intracellular vacuole membranes for the recruitment of specific proteins, which are crucial for remodelling the lipid composition and ensuring bacterial replication.	Depletion of PI4KIIα decreases the production of vacuoles necessary for *Chlamydia trachomatis* replication.	[34]
*Legionella pneumophila*	PI4P recruitment to ensure bacterial replication.	PI4KIII inhibition leads to a significant reduction in pathogen proliferation.	[31]
*Listeria monocytogenes*	Cellular invasion is PI4KIIα dependent.	Suppression of PI4KIIα results in a decrease in the invasion of *Listeria monocytogenes*	[33]
*Streptococcus pyogenes* (*GAS*)	Recruitment of PI4Ks to regulate PI4P levels results in the formation of GAS‐containing autophagosome‐like vacuoles (GcAVs) and the subsequent elimination of GAS.	—	[35]
*Babesia* spp.	PI4Ks are important for *Babesia* replication.	Inhibition of PI4K has been shown to result in potent activity against *Babesia* species.	[37]

### The latest developments in the field of PI4K inhibitors

Notably, PI4K catalyses the phosphorylation of phosphatidylinositol to generate PI4P. Accordingly, investigation of PI4K inhibitors, particularly ATP‐competitive inhibitors, has become important. These compounds inhibit PI4K activity by competing with ATP for binding at the kinase active site, thereby blocking downstream signalling pathways. Numerous PI4K inhibitors have been developed and investigated; however, few specifically target type II PI4Ks. In contrast, PI4KIIIα inhibitors have received greater attention due to the demonstrated role of PI4KIIIα in the replication of cardioviruses such as encephalomyocarditis virus and hepatitis C virus (HCV), as identified through studies of the HCV life cycle [39]. As a result, PI4K inhibitors have been explored as potential antiviral agents. Nevertheless, certain compounds have exhibited notable toxicity in murine models, raising concerns about their clinical applicability [40]. Despite the toxicity associated with some antiviral applications, alternative therapeutic uses for PI4K inhibitors have emerged. One such compound, PI‐273, has been investigated for its effects on breast tumour growth and has shown potential as an anticancer agent [41].

Phosphatidylinositol 4‐kinase beta (PI4Kβ) plays a crucial role in viral replication and was initially identified as a component of the viral replication machinery [42]. Subsequent studies confirmed its involvement in the life cycles of various viruses, including SARS‐CoV‐2 [43] and members of the *Picornaviridae* family [42, 44], leading to its extensive investigation as a therapeutic target. Several PI4Kβ inhibitors have been studied for their antiviral potential, each exhibiting different mechanisms of action. The first inhibitor identified, PIK93, was shown to impair rhinovirus replication [45] and has also been evaluated in the context of neurological disorders, including amyotrophic lateral sclerosis [46]. Additional compounds, such as PI4KIIIβ‐IN‐9 and PI4KIIIβ‐IN‐10, have demonstrated antiviral activity against HCV [47]. Moreover, KR‐27370 has been proposed as a broad‐spectrum antiviral agent, with reported efficacy against *Picornaviridae* viruses [48].

Among the various classes of PI4Kβ inhibitors, the pyrazolo[1,5‐a]pyrimidines are particularly noteworthy. Enviroxime, the first compound of this class to enter clinical trials, exhibits antiviral activity against enteroviruses [49] and has also been investigated for the treatment of HCV infections [50]. A derivative compound, T‐00127‐HEV1, has since been developed and demonstrates improved selectivity compared to enviroxime [51]. GSK3923868 represents another promising example. It was developed as an inhalable PI4Kβ inhibitor for the treatment of human rhinovirus infections in patients with chronic obstructive pulmonary disease and is currently under clinical evaluation [52]. Similarly, CUR‐N399 has been investigated for its therapeutic potential in hand‐foot‐and‐mouth disease and has exhibited efficacy against several members of the *Picornaviridae* family [53]. In addition, recently studied compounds such as KR‐26549 and KR‐27370 have demonstrated high potency and selectivity, positioning them as strong candidates for the development of antiviral therapies targeting human rhinoviruses and other enteroviruses [48].

Another promising class of PI4Kβ inhibitors is the imidazo[1,2‐b]pyridazines, exemplified by MI4, which has shown excellent antiviral activity against viruses from the *Picornaviridae* and *Flaviviridae* families. In contrast, the related compound MI356 displays substantially reduced efficacy against positive‐sense RNA viruses, including HRV1, CVB3 and HCV genotypes 1b and 2a [54].

Also receiving attention are bithiazoles, initially developed as multitarget agents in a PI4Kβ‐directed drug development initiative aimed at treating cystic fibrosis and associated enteric infections. These compounds subsequently demonstrated broad‐spectrum antiviral activity, primarily against viruses of the *Picornaviridae*, *Flaviviridae* and *Coronaviridae* families. The most notable example, MR340, exhibits antiviral activity against HRV2 and HRV14 (human rhinoviruses; *Picornaviridae*), Zika virus (*Flaviviridae*) and SARS‐CoV‐2 (*Coronaviridae*) [55]. Further optimisation efforts led to the development of two MR340 derivatives, MR373 and MR370, which showed improved profiles in terms of the balance between selectivity and antiviral potency [56, 57].

Other PI4KIIIβ inhibitors have been explored. Compounds such as UCB9608 and BQR‐695 exhibit antiviral activity against viruses belonging to the *Coronaviridae* family [58]. In addition, GW5074 was developed to specifically inhibit PI4KIIIβ and has been extensively studied in the context of *Coxsackievirus* infection [59]. Although the dual‐isoform inhibitor AL‐9 targets both PI4KIIIα and PI4KIIIβ, it exerts a stronger inhibitory effect on PI4KIIIα, with limited impact on the β isoform [60] (Table 2).

**Table 2:** PI4K inhibitors targeting host cell kinases (PI4KIIIα/PI4KIIIβ).

Inhibitor	Structure^a^	Isoform target	Main indication/activity	Stage of development	References
PIK93	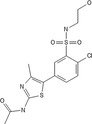	PI4KIIIβ	Antiviral (rhinovirus); investigated for neurological disorders	Preclinical	[45, 46]
PI4KIIIβ‐IN‐9/IN‐10	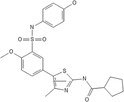	PI4KIIIβ	Antiviral activity against HCV	Preclinical	[47]
Pyrazolo[1,5‐a]pyrimidines (Enviroxime)	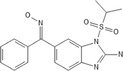	PI4KIIIβ	Enteroviruses and treatment of HCV infections	Early clinical trials	[49, 50]
Pyrazolo[1,5‐a]pyrimidines (T‐00127‐HEV1)	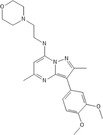	PI4KIIIβ	Enteroviruses and treatment of HCV infections, with improved selectivity when compared to enviroxime	Preclinical	[51]
Pyrazolo[1,5‐a]pyrimidines (GSK3923868)	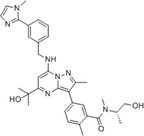	PI 4KIIIβ	Human rhinovirus infections and in patients with chronic obstructive pulmonary disease	Clinical (Phase 1b reported for inhalation study)	[52]
Pyrazolo[1,5‐a] pyrimidines (CUR‐N399)	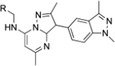	PI4KIIIβ	Hand‐foot‐and‐mouth disease and efficacy against Picornaviridae family	Advanced preclinical; clinical candidate	[53]
Pyrazolo[1,5‐a]pyrimidines (KR‐27370/KR‐26549)	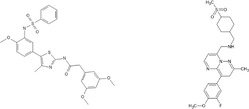	PI4KIIIβ	Human rhinoviruses and other enteroviruses	Preclinical	[48]
Imidazo[1,2‐b]pyridazines (MI4)	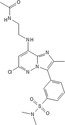	PI4KIIIβ	Viruses from Picornaviridae and Flaviviridae families	Preclinical	[54]
Imidazo[1,2‐b]pyridazines (MI356)	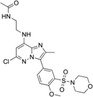	PI4KIIIβ	Viruses from Picornaviridae and Flaviviridae families	Preclinical	[54, 55]
Bithiazoles (MR340)	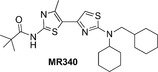	PI4KIIIβ	Broad‐spectrum antiviral activity, focused on HRV2, HRV14 and SARS‐CoV‐2	Preclinical	[55]
Bithiazoles (MR370/MR373, or 2 g/2a, respectively)	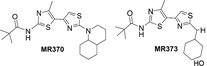	PI4KIIIβ	MR340 derivatives demonstrated improved profiles	Preclinical	[56, 57]
UCB9608	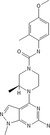	PI4KIIIβ	Antiviral activity against Coronaviridae family	Preclinical and advanced lead (physicochemical properties)	[28]
BQR‐695	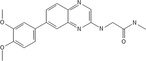	PI4KIIIβ	Antiviral activity against *Coronaviridae* family	Preclinical and research compound	[28]
GW5074	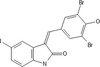	PI4KIIIβ	Activity against coxsackievirus infection	Preclinical and research tool	[58]
AL‐9	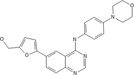	PI4KIIIα and PI4KIIIβ, stronger effect on PI4KIIIα	Antiviral (HCV)	Preclinical and research compound	[59]
PI‐273	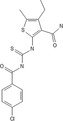	PI4KIIIα	Potential anticancer effects on breast tumour growth	Preclinical and tool compound	[41]

^a^ The molecular structures were drawn using PubChem (https://pubchem.ncbi.nlm.nih.gov//edit3/index.html) and ChemSketch (https://www.acdlabs.com/resources/free‐chemistry‐software‐apps/chemsketch‐freeware/).

Following the identification of *Plasmodium* PI4Ks as targets of imidazopyridine compounds [36], interest in PI4K inhibitors for the treatment of malaria and *Cryptosporidium* infections has grown. Among these, the imidazopyrazine compound KDU691 has shown potent antimalarial activity [60]. Other compounds such as MMV390048 and UCT943 have also demonstrated efficacy against *Plasmodium* species [61, 62]. In addition, KAI715, a compound with PI4K inhibitory activity, has been reported to impair parasite growth [61].

Subsequent studies, including that of Aniweh *et al*. [63], have reinforced the potential of PI4K inhibitors, such as KDU691, as promising pharmacological targets against *Plasmodium* species, particularly *P. falciparum* and *P. ovale*. These findings support the continued evaluation of PI4K‐targeting compounds for the development of next‐generation antimalarial therapies. AZD0156 (MMV1580483), for example, has been identified as a potent and selective inhibitor of *P. falciparum* PI4Kβ, demonstrating promising *in vitro* activity across multiple parasite developmental stages [64].

Further candidates, including KDU731 and KDU691, have been investigated for their potential to treat *Cryptosporidium* infections [65]. In particular, KDU731 demonstrated potent *in vitro* parasiticidal activity against *Cryptosporidium parvum* and had a favourable safety profile in host cells. It showed superior efficacy compared to other tested agents, including nitazoxanide and paromomycin [66]. Furthermore, the compound EDI048, a selective PI4K inhibitor, was designed based on rational principles and has demonstrated potent parasiticidal activity against *Cryptosporidium* spp. Notably, EDI048 is a gut‐restricted agent, which limits systemic exposure and reduces the risk of off‐target effects. The efficacy of the drug has been demonstrated in preclinical models of paediatric enteric cryptosporidiosis [67]. (Table 3).

**Table 3:** PI4K inhibitors targeting parasite kinases (*Plasmodium*, *Cryptosporidium* spp).

Inhibitor	Structure^a^	Parasite of interest	Activity/disease	Stage of development	References
KDU691	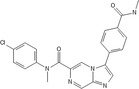	PI4K (*Plasmodium falciparum*)	Antimalarial	Preclinical	[60, 63]
MMV390048	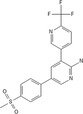	PI4K (*Plasmodium* spp.)	Antimalarial and activity against babesiosis	Clinical (Phase I/II); early human studies–MMV	[38, 61, 62]
UCT943	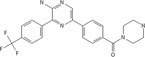	PI4K (*Plasmodium* spp.)	Antimalarial	Preclinical	[61]
RKI‐1447/Silmitasertib	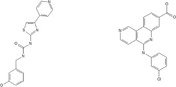	PI4K and other kinases (*Plasmodium falciparum*)	Antimalarial and Oncology	Virtual screening; preclinical for RKI‐1447 And Silmitasertib are in clinical stage (phase1/2)	[68]
KAI715	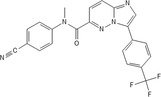	PI4K (*Plasmodium falciparum*)	Antimalarial	Preclinical	[61]
AZD0156 (MMV1580483)	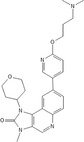	PI4Kβ (*Plasmodium falciparum*)	Antimalarial	Clinical (for ATM) and still experimental (antimalarial)	[64]
2,8‐disubstituted‐1,5‐naphthyridines	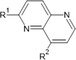	PI4K + haemozoin (*Plasmodium falciparum*)	Antimalarial	Preclinical	[69]
KDU731	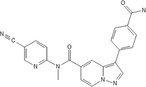	PI4K (*Cryptosporidium parvum*)	Cryptosporidiosis	Preclinical; Advanced Candidate	[65, 66]
EDI048	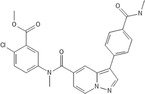	PI4K (Cryptosporidium spp.)	Paediatric cryptosporidiosis	Early Clinical; Candidate	[67]

^a^ The molecular structures were drawn using PubChem (https://pubchem.ncbi.nlm.nih.gov//edit3/index.html) and ChemSketch (https://www.acdlabs.com/resources/free‐chemistry‐software‐apps/chemsketch‐freeware/).

## Conclusion

- PI4Ks are central regulators of key cellular processes such as vesicular trafficking, Golgi maintenance and intracellular signalling. These enzymes play essential roles not only in normal cellular physiology but also in the biology of various pathogens. Numerous studies have shown that viruses and bacteria exploit host PI4Ks to facilitate intracellular replication. From the point of view of the pathogens, the inhibition of parasite PI4Ks led to their impairment, thus highlighting the potential of PI4Ks as attractive targets for the development of antimicrobial therapies.
- Although the development of PI4K inhibitors has shown promise, several challenges remain, particularly in achieving sufficient selectivity, minimising toxicity and ensuring adequate bioavailability.
- Selective inhibition of PI4Ks represents a compelling therapeutic strategy against a broad spectrum of pathogens. Continued research into the structural, functional and pharmacological properties of PI4Ks is critical for the development of safe and effective therapeutic agents targeting these enzymes.

## Author contributions

ACM and DB conceptualised and designed the article; ACM, GMA and APB performed research; ACM, GMA, APB and DB wrote the paper. All authors were involved in drafting and revising the manuscript.
